# The use of induced pluripotent stem cells in domestic animals: a narrative review

**DOI:** 10.1186/s12917-020-02696-7

**Published:** 2020-12-08

**Authors:** Rachel A. Scarfone, Samantha M. Pena, Keith A. Russell, Dean H. Betts, Thomas G. Koch

**Affiliations:** 1 Department of Biomedical Sciences, Ontario Veterinary College, University of Guelph, 50 Stone Road East, Guelph, Ontario N1G 2W1 Canada; 2 Department of Physiology and Pharmacology, The University of Western Ontario, London, Ontario N6A 5C1 Canada

**Keywords:** Induced pluripotent stem cells, Domestic species, Veterinary medicine, Production, Characterization, Disease modelling, Disease treatment

## Abstract

Induced pluripotent stem cells (iPSCs) are undifferentiated stem cells characterized by the ability to differentiate into any cell type in the body. iPSCs are a relatively new and rapidly developing technology in many fields of biology, including developmental anatomy and physiology, pathology, and toxicology. These cells have great potential in research as they are self-renewing and pluripotent with minimal ethical concerns. Protocols for their production have been developed for many domestic animal species, which have since been used to further our knowledge in the progression and treatment of diseases. This research is valuable both for veterinary medicine as well as for the prospect of translation to human medicine. Safety, cost, and feasibility are potential barriers for this technology that must be considered before widespread clinical adoption. This review will analyze the literature pertaining to iPSCs derived from various domestic species with a focus on iPSC production and characterization, applications for tissue and disease research, and applications for disease treatment.

## Background

Induced pluripotent stem cells (iPSCs) are laboratory-developed pluripotent stem cells generated by the reprogramming of differentiated cells [1]. Takahashi and Yamanaka first discovered somatic cells’ capacity for reprogramming in 2006 after forcing differentiated fibroblast cells to ectopically express four transcription factors associated with pluripotency: Oct4, Sox2, Klf4, and c-Myc, collectively referred to as OSKM [1, 2]. iPSCs have since been of interest to researchers in the fields of toxicology, pathology, virology, developmental anatomy and physiology, amongst others [3–5]. iPSCs possess several benefits over other stem cell types such as mesenchymal stromal cells (MSCs) and embryonic stem cells (ESCs). In the context of this review, the term mesenchymal *stromal* cells has been adopted over mesenchymal *stem* cells due to the finite self-renewing property of MSCs that does not support the traditionally recognized self-renewing characteristic of stem cells [6]. The versatility of iPSCs may make them preferential over MSCs that are limited in their differentiation potential due to their multipotent nature [7–9]. ESCs offer a similar versatility to iPSCs as they are both pluripotent, but not without limitations [8]. ESCs can be obtained from in vivo and in vitro produced embryos at the blastocyst stage [10]. However, technical difficulties have interfered with the isolation and use of ESCs, namely in ungulate species and canines [2, 8, 11, 12]. Oocyte collection for in vitro embryo production is an invasive procedure that has prompted ethical considerations. Disposed reproductive material has been the primary source of oocytes in domestic species obtained from meat processing in livestock or ovariohysterectomies in companion animals [13–15]. In vivo protocols may include minimally invasive uterine flushing, often seen in mares [10]. iPSCs provide a more practical alternative to creating ESC-like cells in species where recovery of embryos or in vitro fertilization is difficult or not possible [12]. Unlike ESC lines, autologous iPSC lines can also be produced. This is ideal for transplantation of stem cells and their derivatives as it avoids the immunological complications associated with allogeneic iPSCs. Consequently, iPSCs can be used as an alternative to MSCs and ESCs with the potential for greater research and clinical applicability in domestic species.

**Fig. 1:**
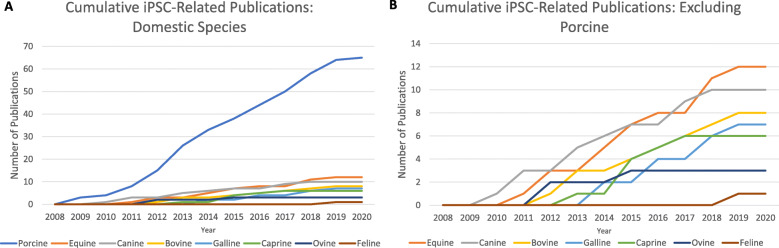
Cumulative iPSC-Related Publications in Domestic Species, January 2008–March 2020. **a** Publications regarding induced pluripotent stem cells from January 2008 to March 2020 in domestic animal species including porcine, equine, canine, bovine, galline, caprine, ovine and feline. Increased interest in iPSC research in domestic animals is demonstrated, particularly in the porcine model. **b** A subset of publications excluding porcine papers to visualize the general positive trend in all other domestic species

While research has focused primarily on human and mice iPSCs, there has been a slow accumulation of iPSC research in domestic animals in the last decade (Fig. 1). iPSC derivation protocols have been developed in species including porcine [16], equine [17], canine [18], bovine [19], galline [20], caprine [21], ovine [22], and feline [23]. Aside from their importance in treating veterinary pathologies, porcine, canine, and equine models have been shown to be valuable for the study and treatment of human disease [24–26]. The purpose of this review is to provide an overview of the literature pertaining to current protocols and applications of iPSCs derived from domestic species. This review will address the topics of the development and use of iPSCs for tissue and disease research, their treatment in domestic animals and the barriers to their production and applications.

## iPSC production and characterization

**Fig. 2:**
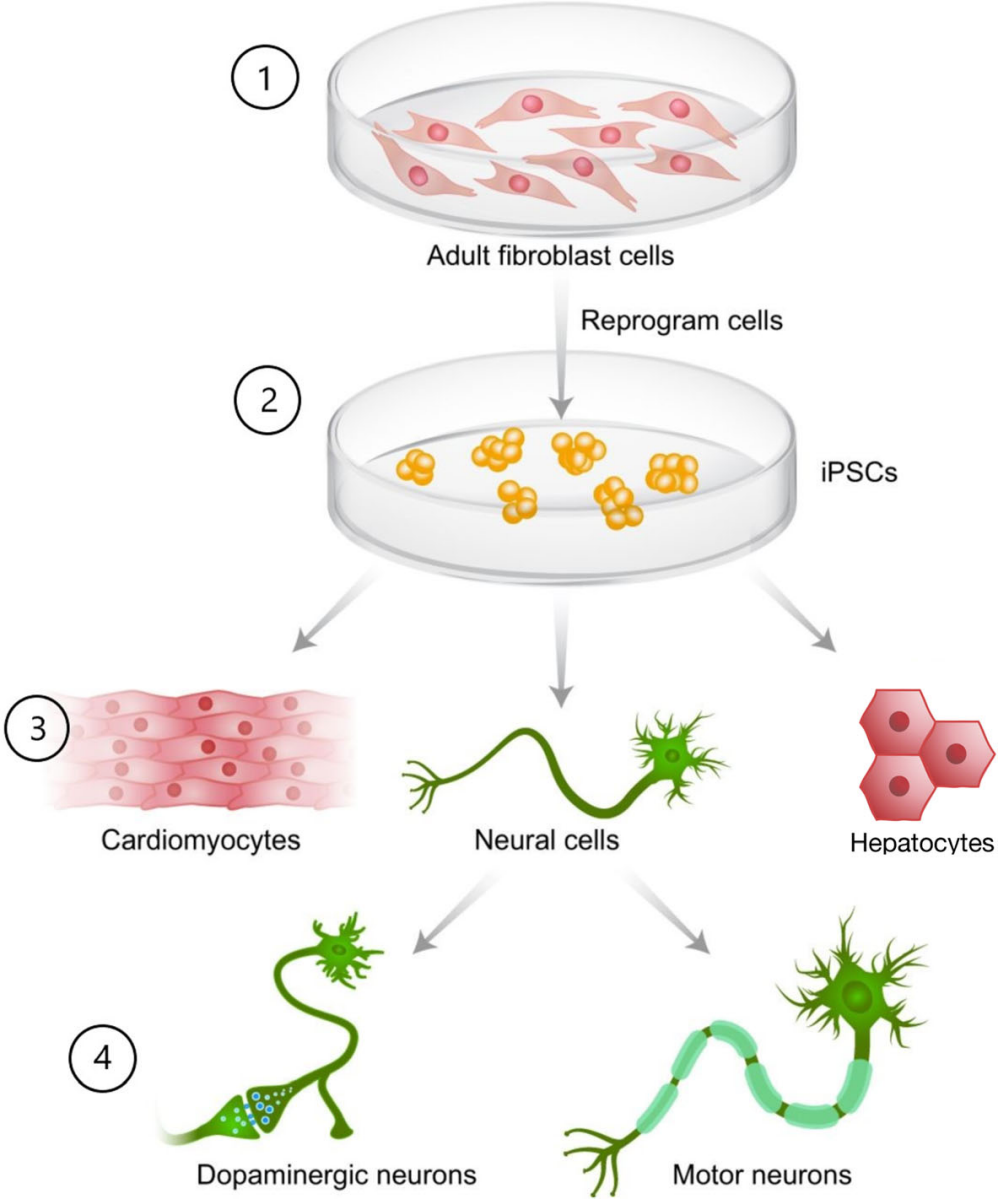
Induced Pluripotent Stem Cell Production and Differentiation. Differentiated cells, e.g. adult fibroblast cells [1], can be reprogrammed via designated reprogramming factors (e.g. Oct4, Sox2, Klf4, and c-Myc), to create iPSCs [2]. Upon exposure to specific differentiation media, iPSCs are capable of differentiating into any cell type of the body, e.g. multipotent neural cells [3]. Under appropriate culture conditions, iPSCs can result in a fully differentiated cell, e.g. a motor neuron [4]. Figure from “Induced pluripotent stem cell model of lysosomal storage disorders,” by Borger DK et al., 2017, Dis Model Mech. 10:691–704, CCBY [28] with minor alterations using Microsoft Word

Yamanaka and colleagues’ discovery of iPSCs originated in mice models, followed closely by their derivation from human fibroblasts [1, 27]. Briefly, mice tail fibroblasts or human dermal fibroblasts were cultured then transduced with retroviral vectors containing expression cassettes of the OSKM reprogramming factors, inducing pluripotency in the transduced cells (Fig. 2). Using these protocols as a base, methods have been adapted in order to produce iPSCs in other species.

iPSCs have been developed from porcine [16], equine [17], canine [18], bovine [19], galline [20], caprine [21], ovine [22], and feline [23] tissue. Successful iPSC production from domestic species was first reported in 2009 by Wu and colleagues in porcine, and the field has since expanded to other species (Fig. 1).

iPSCs have been produced from various donor tissue types, transduction systems, and reprogramming factor combinations. In domestic species, iPSCs have been derived from fibroblasts, MSCs and other somatic cell types including epithelial and testicular cells (Table 1). Tissue sources have been obtained from various developmental stages, namely fetal, neonatal, juvenile, and adult. For simplicity, this review has identified any tissue sources obtained from an animal in utero as fetal and those obtained after birth as adult. Deriving iPSCs from adult somatic cells is generally preferable to embryonic derivation due to a higher abundance of cells, easier collection of cells, and the ability to produce autologous iPSC populations for disease treatment. Donor tissue is then cultured and reprogrammed using viral or non-viral vectors containing the designated reprogramming factors. Viral vectors include lentiviruses, oncoviruses, and Sendai viruses, while non-viral vectors include cDNA vectors, minicircles and transposons (Table 1). The selected reprogramming factors typically include OSKM, but other variations have also been explored. Nanog and Lin28 are commonly used in the literature in addition to OSKM, and a small number of papers report the use of other additional transcription factors, such as TERT, and Tet1 (Table 1). More recently, work has been carried out using microRNAs in combination with other factors to achieve pluripotency induction [19, 29, 67]. MicroRNAs alone have only shown partial reprogramming abilities in domestic animals [61].

**Table 1:** iPSC Production

Origin cell type	Reprogramming system	Reprogramming factors	Reference
***Porcine***			
Fetal fibroblasts	Unspecified retroviral vectors	OSKM	[29–38]
Fetal fibroblasts	Oncoviral vectors	OSKM	[39]
Fetal fibroblasts	Lentiviral vectors	OSKM	[16, 40–46]
Adult sertoli cells	Unspecified retroviral vectors	OSKM	[47, 48]
Adult fibroblasts	Unspecified retroviral vectors	OSKM	[49]
Adult fibroblasts	Lentiviral vectors	OSKM	[24, 50–53]
Adult fibroblasts	Sendai viral vectors	OSKM	[54]
Adult fibroblasts	Lentiviral vectors	OSKM, Nanog, Lin28	[55, 56]
Adult MSCs	Lentiviral vectors	OSKM, Nanog, Lin28	[57]
Adult MSCs and fibroblasts	Lentiviral vectors	OSKM	[58]
Adult fibroblasts and bone marrow cells	Lentiviral vectors	OSKM, Nanog, LIN28	[59]
Fetal fibroblasts	*PiggyBac* transposon	OSKM	[60]
Fetal fibroblasts	Lentiviral vectors	miR-302 s	[61]
Fetal fibroblasts	Unspecified retroviral vectors	OSKM	[62]
Fetal fibroblasts	Episomal plasmids	Oct3/4, Sox2, Klf4, I-Myc	[63]
Adult fibroblasts	Lentiviral vectors	OSKM, Nanog, LIN28	[64]
Fetal fibroblasts	Unspecified retroviral vectors	OSKM, mTet3, Tet1, Kdm3a	[65]
Fetal fibroblasts	Lentiviral vectors	OSKM, Nanog, LIN28	[66]
Fetal fibroblasts	Unspecified retroviral vectors	OSKM, miR-106a-363, and miR-302	[67]
Fetal fibroblasts	Lentiviral vectors	OSKM, or OSKM, Tbx3, Nr5a2	[68]
Fetal fibroblasts	Sleeping Beauty transposon	OSKM, Nanog, LIN28	[69]
Fetal fibroblasts	Unspecified retroviral vectors	OSKM, TERT	[70]
Fetal and adult fibroblasts and MSCs	Unspecified retroviral vectors and lentiviral vectors	OSKM	[71]
Adult fibroblasts	Unspecified retroviral vectors	OSKM	[72]
Fetal fibroblasts	Sleeping Beauty transposon	OSKM, Nanog, LIN28	[73]
Fibroblasts	Sleeping Beauty transposon	OSKM	[74]
GALT-KO fibroblasts	Lentiviral vectors	OSKM, Nanog, LIN28	[75]
Adult fibroblasts	Lentiviral vectors	OSKM, Nanog, LIN28	[76]
Adult MSCs	Lentiviral vectors	OSKM	[77]
Fetal fibroblasts	Episomal vectors	Oct4, Sox2, Klf4	[78]
Fetal MSCs	Unspecified retroviral vectors	Oct4, Klf4	[79]
Fetal fibroblasts	Lentiviral vectors	OSKM, Nanog	[80]
Adult fibroblasts	Unspecified retroviral vectors	OSKM	[81]
***Equine***			
Fetal fibroblasts	*PiggyBac* transposon	OSKM	[17]
Adult fibroblasts	Unspecified retroviral vectors	Oct4, Sox2, Klf4	[82]
Adult fibroblasts	Unspecified retroviral vectors	OSKM	[83]
Adult fibroblasts	*PiggyBac* transposon	OSKM	[84, 85]
Adult keratinocytes	Unspecified retroviral vectors	OSKM	[86]
Adult MSCs	Lentiviral vectors	OSKM	[87]
***Canine***			
Fetal fibroblasts	Unspecified retroviral vectors	OSKM	[18]
Fetal fibroblasts	Sendai viral vectors	OSKM	[88]
Fetal fibroblasts	Lentiviral vectors	OSKM	[89]
Adult fibroblasts	Unspecified retroviral vectors	OSKM	[90]
Adult fibroblasts	Sendai viral vectors	OSKM	[12]
Adult fibroblasts	Lentiviral vectors	OSKM	[91]
Adult MSCs	Lentiviral vectors	OSKM	[92]
Adult MSCs	Unspecified retroviral vectors	OSKM	[93]
***Bovine***			
Adult testicular cells	Electroporation	Oct4	[94]
Fetal fibroblasts	Unspecified retroviral and lentiviral vectors	OSKM, Nanog	[95]
Fetal fibroblasts	Lentiviral vectors	OSKM	[43]
Fetal fibroblasts	Unspecified retroviral and lentiviral vectors	OSKM, Nanog, Lin28	[96]
Fetal fibroblasts	Unspecified retroviral and lentiviral vectors	OSKM, Nanog, Lin28, SV40TAg, TERT	[96]
Adult neural stem cells	Lentiviral vectors	miR-302, miR-367	[19]
Adult epithelial cells	Oncoviral vectors	OSKM	[97]
Adult fibroblasts	Oncoviral vectors	OSKM	[97]
Fetal fibroblasts	Unspecified retroviral vectors	OSKM	[98]
Fetal fibroblasts	Lentiviral vectors	OSKM	[99]
***Galline***			
Fetal fibroblasts	Nonviral minicircle DNA	OSKM, Nanog, LIN28	[20]
Fetal fibroblasts	Lentiviral vectors	OSKM, LIN28	[100]
Fetal fibroblasts	Lentiviral vectors	OSKM, Nanog, LIN28	[101]
Adult fibroblasts	*piggyBac* transposon	M3O, Sox2, Klf4, c-Myc, LIN28, Nanog	[102]
Fetal fibroblasts	Oncoviral vectors	OSKM	[103]
***Caprine***			
Fetal fibroblasts	Lentiviral vectors	OSKM, Nanog, Lin28	[11]
Adult fibroblasts	Lentiviral vectors	OSKM	[104]
Fetal fibroblasts	cDNA vectors	OSKM	[105]
Fetal fibroblasts	Lentiviral vectors	OSKM, PRMT5	[106]
Fetal fibroblasts	Lentiviral vectors	OSKM	[21, 107]
***Ovine***			
Fetal fibroblasts	Unspecified retroviral vectors	OSKM	[108, 109]
Fetal fibroblasts	Oncoviral vectors	OSKM	[110]
***Feline***			
Fetal fibroblasts	Lentiviral vectors	OSKM, Nanog	[23]

Following presumed iPSC production, colonies can be analyzed via morphological assessment to select for colonies with the most potential in reprogramming cells to an undifferentiated state. Non-invasive morphological assessment also provides insight into the developmental competence and homogeneity of iPSC colonies. Traditionally, iPSC colonies resemble ESC colonies with well-defined borders and tightly packed cells. More specifically, dome-shaped and flattened colonies are indicators of naïve and primed pluripotency, respectively [111]. Cells in these colonies are expected to have a large nucleus and little cytoplasm [112]. Naïve pluripotency is recognized by characteristic molecular features of the pre-implantation mouse embryonic stem cell, whereas primed pluripotency resembles stem cells of the post-implantation mouse epiblast [113]. Naïve pluripotent stem cells are identified by X chromosome reactivation in females, dependency on leukemia inhibitory factor (LIF) and receptivity to BMP4 to maintain pluripotency, and the transition to a more differentiated state in response to FGF2 and ACTIN/TGFB signalling [58, 114, 115].

Putative iPSCs must then undergo a series of tests to confirm pluripotency (Table 2). In domestic species, pluripotency is often confirmed by the endogenous expression of pluripotency markers, and the formation of in vitro embryoid bodies and in vivo teratomas containing cell types derived from all three germ layers [118]. Chimera formation with germ-line transmission is a less commonly used method in domestic species (as demonstrated in Table 2), but is deemed the gold standard for validating stem cell pluripotency [119].

**Table 2:** iPSC characterization (specific to articles that investigated chimerism)

Origin cell type	Suggested pluripotent state	iPSC characterization criteria					Reference
		Pluripotency markers	Embryoid bodies	Teratomas	Chimeras	Germline transmission	
***Porcine***							
Adult MSCs	Primed	Yes	Yes	Not tested	Yes	Yes	[57, 116]
Fetal fibroblasts	Naïve	Yes	Yes	Yes	Yes; limited to blastocyst	Not tested	[117]
Fetal fibroblasts	Naïve	Yes	Yes	No	Yes, limited to fetus	Not tested	[40]
***Ovine***							
Fetal fibroblasts	Primed	Yes	Yes	Yes	Yes; but low contribution at birth	Not tested	[108]
***Galline***							
Fetal fibroblasts	Primed	Yes	Yes	Not tested	Yes	Not tested	[20]

Although formation of embryoid bodies and teratomas are successful in the majority of papers referenced in this review, many publications lack complete pluripotent characterization of their produced cell lines. Consequently, this review uses the term iPSCs broadly to describe both bona fide iPSCs and iPSC-like cells as some primary research lacks sufficient iPSC characterization to confirm true pluripotency. A common occurrence among studies is the incomplete silencing of exogenous transcription factors. These cell lines may not be truly pluripotent, but are reliant on transgenes to maintain pluripotency. The use of epigenetic modifiers has previously been shown in human and mice cells to increase transgene silencing while maintaining endogenous pluripotency factor expression; results that have now been replicated in the porcine model [65]. This technique could be promising to alleviate these issues, although more work will be required.

As previously mentioned, the gold standard for validating stem cell pluripotency is via chimera formation with germ-line transmission. Chimera formation is defined by the heterogeneous cell population of an early embryo following the injection of iPSCs into the blastocyst. This confirmation method requires iPSCs to integrate into the developing embryo and contribute to all three germ layers and, potentially, germ cells. To confirm germ-line transmission, chimeras are mated with non-chimeras and offspring are assessed for iPSC contribution [116]. Previous research has suggested that the feasibility of chimerism and germ-line transmission is greatly improved with the use of naïve pluripotent stem cells, as opposed to primed pluripotent stem cells [2, 40]. Although porcine naïve iPSC-like cells have been reported, there is little evidence of germline transmission. Analysis of the literature suggested that the generation of chimeras in livestock species is difficult to achieve [20]. Injection of generated iPSCs into an embryo often resulted in limited incorporation [40, 108] and the resulting offspring often were not chimeric [40, 108, 117]. Significant variation in iPSC integration has been shown [19, 112]. To the best of our knowledge, West and colleagues remain the only researchers to successfully realize germline transmission of iPSCs in a domestic species (i.e. porcine) [116]. iPSCs were also tested in cloning transgenic tissue [55, 104] and genomic incorporation of transgenes [60]. ﻿It was found that iPSCs could be derived from transgenic organisms, specifically genetically modified pigs designed for xenotransplantation [55]. However, limited developmental potential of embryos past the blastocyst stage was observed [60]. Hence, analysis of germline transmission is currently not feasible in such species. In moving forward with clinical applications of iPSCs in either human or veterinary medicine, being able to truly define cells as iPSCs will be crucial for standardization, quality, and safety assurances.

## Tissue and disease research

iPSCs have the potential to be valuable tools for tissue and disease modelling. In vitro differentiation of iPSCs has allowed for study of the developmental processes and pathologies of tissues and may allow for preclinical testing of therapeutic drugs for veterinary and human medicine. With regard to drug screening, there has been success in human and mouse iPSC research in using differentiated iPSC lines to model disease and conduct high-throughput screening of small molecules for their effects on disease progression [120]. This technique allows for testing of potential therapeutics against disease-genotype cells specific to an individual or species without the need for interspecies comparisons or excessive lab animal use. Differentiation into specific cell types has been noted many times in the literature in porcine, equine, canine, galline, and bovine models, which are described below. Although characterization of these differentiation cells is demonstrated by physiological, genetic, or metabolic capacities of cell lines, the degree of differentiation varies from progenitor cells (e.g. neural progenitors [41, 76, 121]), to fully differentiated cell types (e.g. skeletal myocytes [122]). Domestic animal diseases are abundant and have negative health effects for consumers of agricultural animal by-products [123–126]. Unfortunately, the use of stem cells for research on livestock disease is novel and presently limited in number. The prolonged self-renewing characteristic of iPSCs supports their use in the study of physiology, disease pathology, drug toxicity and vaccine development in domestic species. A summary of veterinary animal iPSC research can be found in Table 3.

**Table 3:** iPSC publications relating to tissue and disease modelling

Origin cell type	Differentiated cell type	Tissue or disease target	Outcome	Reference
***Porcine***				
Fibroblasts	Neural Rosettes, neural crest-like cells, and peripheral sensory neural-like cells	Neural tissue	Indications of neural differentiation by the upregulation of sensory neuron genes and peripheral neuron markers	[121]
Adult fibroblasts	Neurons, astrocytes, and oligodendrocytes	Neural tissue	Indications of neural differentiation by the presence of mature neural markers and morphology of neurons, astrocytes, and oligodendrocytes; indications of further differentiation into motor neurons.	[76]
Fetal fibroblasts	Neural progenitor cells	Neural tissue	Production of neural progenitor cells with expression of neuronal markers	[41]
Fetal fibroblasts	Endothelial cells	Endothelial tissue	Production of endothelial cells with morphological and functional properties	[30]
Fetal fibroblasts	Hepatocyte-like cells	Liver tissue	Production of differentiated cells characteristic of hepatocytes by functional properties	[78]
Adult fibroblasts	Hepatocyte-like cells	Liver tissue	Production of differentiated cells characteristic of hepatocytes functional properties	[50]
Fetal fibroblasts	Vascular smooth muscle cells	Muscle tissue	Production of vascular smooth muscle cells capable of forming 3D scaffold-free tissue rings	[42]
***Equine***				
Adult fibroblasts	Cortical neurons	West Nile Virus (WNV) and Murray Valley Encephalitis (MVEV)	Successful infection of functional eiPSC-derived neurons by WNV and MVEV	[127]
Adult keratinocytes	Cholinergic motor neurons	Motor Neurons	Production of functional neurons capable of generating action potentials	[86]
Fetal and adult fibroblasts	Tenocytes	Tendons	Formation of three-dimensional artificial tendons	[128]
Fetal fibroblasts	Skeletal myotubes	Muscle Tissue	Formation of eiPSC-derived muscle fibers with electrophysiological function	[122]
Adult fibroblasts	Osteoblasts	Bone	Formation of eiPSC-derived bone tissue capable of secreting hydroxyapatite and calcium matrix	[129]
Adult keratinocytes	Primary keratinocytes	Epidermal wounds	Creation of artificial tissues for potential skin graft applications	[84]
***Canine***				
Adult fibroblasts	MSCs	Cartilage and Bone Tissue	Formation of three-dimensional chondrogenic and osteogenic cultures	[130]
Fetal fibroblasts	Mature megakaryocytes	Thrombocytopenia	Production of cells capable of releasing functional platelets upon signaling induction	[131]
***Bovine***				
Adult testicular cells	N/A	Phthalate ester exposure	Significant reduction in androgen expression and increase in apoptosis	[94]
Adult epithelial cells	Mammary epithelial-like cells	Mammary tissue	Indication of mammary phenotype for iPSCs cultured with progesterone	[97]
***Galline***				
Fetal fibroblasts	N/A	Goose influenza H5	Incorporation of replication-incompetent virus into iPSCs	[100]
Fetal fibroblasts	N/A	Newcastle disease virus (NDV)	Successful infection of iPSCs with NDV; viable iPSCs exhibited increased tolerability	[132, 133]

### Porcine

Porcine iPSCs (piPSCs) have been differentiated into several cell types for research purposes. Currently, they have been used in the production of neural progenitor cells [41, 76, 121], endothelial cells [30], myotubes [134], hepatocytes [50, 78], and vascular smooth muscle cells (VSMCs) [42]. VSMCs in particular have been applied to scaffolds for implantation into immunodeficient mice and successfully formed 3D scaffold-free tissue rings [42].

### Equine

Equine iPSCs (eiPSCs) have been differentiated into several cell and tissue types for disease modelling including neurons [86, 127], tendons [128], myotubes [122], and osteoblasts [129]. Functional eiPSC-derived neurons have been produced and were capable of firing action potentials in vitro via functional calcium channels [86]. One paper reported the observation of neurospheres with axonal outgrowths connecting adjacent cells [127]. This paper studied the potential for neurospheres to model West Nile virus (WNV) and Murray Valley encephalitis virus (MVEV), infectious, neurotropic equine diseases [127]. iPSC-derived neurons were successfully infected by WNV and MVEV, which could allow for future research to study mechanisms of these and other infectious diseases and neuropathic conditions.

Musculoskeletal tissue is a major system that would benefit from eiPSC modelling due to the frequency of injuries in competing horses. Artificial tendons derived from iPSCs have been attempted and although two-dimensional assays showed matrix contraction and appropriate gene expression, three-dimensional assays failed to generate functional artificial tendons. ESCs were shown to more efficiently produce functional tendons [128]. Further study is required here as this could be a promising area of regenerative medicine if eiPSC-derived tendons can be improved. Using fibroblast-derived eiPSC lines, researchers induced differentiation into myocytes, the functional unit of muscles. Myotubes demonstrated intracellular calcium release following membrane depolarization [122]. Lastly, functional eiPSC-derived osteoblasts have been reported. These cells expressed genetic markers of osteoblasts and were shown to produce hydroxyapatite and calcium matrices, highly specific characteristics of bone tissue [129]. Artificial production of bone may allow for study of bone physiology and diseases but may also benefit veterinary treatment of fractures and other pathologies.

Wound management is a common problem in equine medicine, and skin grafting, the ideal treatment, is often not possible due to a low supply of donor tissues [135]. One paper described a protocol where eiPSCs were differentiated into keratinocytes (eiPSC-KCs) to produce skin grafts. The eiPSC-KCs were likened to both progenitor and primary keratinocyte-like cells, potentially indicative of epidermal basal stem cell identity, ideal for in vivo wound management [84].

### Canine

MSCs derived from canine iPSCs (ciPSCs) have been proposed as an intermediate stage to developing canine models of musculoskeletal tissues through chondrogenic and osteogenic pathways [130]. ciPSCs were differentiated into MSCs and subsequently differentiated into chondrocytes and osteoblasts in three-dimensional hydrogel culture conditions. Researchers proposed these three-dimensional cultures as effective models for studying canine osteoarthritis in order to develop MSC-based therapies and further model human degenerative joint disease [130].

A novel protocol has been published to generate functional canine platelets to treat thrombocytopenia, a canine and human clotting disorder. ciPSCs were differentiated into mature megakaryocytes which could be induced to release functional platelets [131]. This could serve as an alternative treatment to blood transfusion, the only effective therapy currently available.

### Galline

Galline iPSCs (giPSCs) have been used in studying viral infection and replication [100, 132, 133]. Newcastle disease (NDV) is a common avian viral disease often found in domestic poultry [132]. Studies have demonstrated that giPSCs are capable of NDV infection [132, 133], and that viable cells displayed increased tolerability but not immunity to the virus [133]. giPSCs could also be used to produce replication-incompetent viruses, such as the highly pathogenic H5 avian influenza viruses [136]. Replication-incompetent viruses were produced with the ﻿goose influenza H5 gene and were incorporated into giPSCs. Using these cells, the virus was further transduced into a bladder cancer-derived cell line and could be inactivated by formaldehyde [100]. The use of giPSCs for vaccine production may be beneficial over chick embryos or eggs due to a decreased risk of contamination [100]. These results suggest that giPSCs have the potential to produce inactive viruses for vaccine production.

### Bovine

Limited disease modelling has been observed with bovine iPSCs (biPSCs); however, current efforts have demonstrated their potential application in toxicological studies to elucidate the effects of toxic environmental compounds. Cattle can be used to investigate the negative effects of environmental endocrine disrupting compounds (EDCs) on humans and livestock species as the potential for harmful chemicals leaching into waterways and soils has become a prominent concern [137]. Despite a lack of clinical evidence, it has been proposed that EDCs can affect the reproductive functioning of cattle, greatly impacting agricultural production [138]. Bovine iPSCs have been applied to research the EDCs phthalate esters [94]. It was found that phthalate esters significantly downregulated androgen receptors of iPSCs, which supported apoptosis [94]. Such studies introduced biPSCs as a feasible tool in studying the effects of endocrine disruptors and other chemicals on cell populations. biPSCs have also been differentiated into epithelial-like cells that phenotypically resembled mammary cells [97]. These cells could further be investigated for their application in tissue regeneration for oncology patients who have undergone a mastectomy.

## Disease treatment

Although research of specific pathologies is generally limited to single publications, the use of iPSCs to treat diseases and injuries in animals is growing and will likely be integrated into veterinary practice in the future. The field of domestic animal regenerative medicine may also provide models for human pathologies. Stem cell research that was once conducted on rodents is now growing in dogs and pigs [139, 140], species shown to be better models for human disease [25, 139, 140]. Table 4 summarizes the current research for iPSC-based treatments in domestic animals, which for the purpose of this review, includes all in vivo applications of iPSCs and their derivatives.

**Table 4:** iPSCs for Disease Research

Disease Target	Origin Cell Type	Differentiated Cell Type	Route of Administration	Outcome	Reference
***Porcine***					
Osteoporosis	Fibroblasts	Osteoblast-like cells	Local cell transplantation	Significant improvement in bone structures at transplanted site; maintenance of bone structures locally	[141]
Osteochondral damage, osteoarthritis	Adult fibroblasts	piPSC-like cells	Direct pellet transplantation	Cartilage regeneration; no tumor formation	[24]
Chronic myocardial infarction	Adult fibroblasts	piPSCs	Direct injection	Integration of iPSCs into cardiac muscle without differentiation; potential contribution to angiogenesis	[51]
Acute myocardial infarction	Adult fibroblasts	piPSCs	Direct injection	Significant decrease in infarcted area; improvement in local function and perfusion	[142]
Myocardial infraction	Adult MSCs	Endothelial cells (ECs)	Local injections	Improved function and an increase in the number of capillaries in the peri-infarct area; no significant changes in infarct area size.	[77]
Chronic spinal cord injury	Adult fibroblasts	Neural precursor cells (NPCs)	Bilateral syngeneic grafts	Long-term immune tolerance of NPCs; integration into and beyond grafted region	[54]
Retinal damage	Fetal fibroblasts	Rod photoreceptors	Local injection	Integration into damaged porcine neural retina	[78, 143]
***Equine***					
Muscle injury	Adult MSCs	eiPSCs	Intramuscular injection	Partial muscle regeneration; in vivo differentiation of eiPSCs into myofibers at the injury site	[87]
Musculoskeletal injury	Adult MSCs	MSCs	Injection into lesion	Improvements in clinical conditions for injuries including fractures, tendonitis, osteochondrosis, and osteoarthritis	[144]
***Canine***					
Hind limb ischemia	Adult MSCs and fibroblasts	Endothelial cells	Local injections	Successful engraftment in the ischemic limb; significant improvement of vascularization locally	[92]
Cardiac infarction	Adult MSCs and fibroblasts	Endothelial cells	Local injections	Successful engraftment locally; improvement in cardiac contractility	[92]

### Porcine

Pigs are the most frequently used model of disease in domestic species. Porcine iPSCs have been employed in the study of tissue regeneration in bone [24, 141], muscle [51, 142], and nervous tissue [54, 143]. The findings in a majority of the articles published confirms that piPSCs are capable of integrating into tissue at the site of implantation [142, 143] and are capable of cueing endogenous pathways to upregulate [51], thus improving conditions at the site of tissue damage or death.

In a study of bone regeneration, piPSC-derived osteoblast-like cells were able to improve the trabecular and cortical bone structures of fractured tibias [141]. In a similar study, partial tibial cartilage regeneration at the transplantation site was observed with the regenerated cartilage originating from iPSCs [24].

Other studies examined the beneficial treatment effect of iPSCs on chronic myocardial ischemia [51] and infarction [142]. Regenerative therapy of cardiac tissue in porcine models involved direct injection of undifferentiated piPSCs into myocardium [51, 142]. The treatment was found to significantly decrease the infarction area, decrease regional perfusion, and increase angiogenesis with local incorporation of piPSCs into myocardium and blood vessel without tumor formation [142]. A similar study found small tumor formations that eventually arrested in growth [51]. While the research suggested variations in the grafting capabilities of these piPSCs into host tissues, there was an identified increase in smooth muscle actin, indicating piPSCs interact in some form with host tissue. In brief, piPSCs have been shown to contribute to myocardium regeneration.

piPSCs were also applied to regenerate nerve tissue. Researchers differentiated porcine iPSCs into neural progenitor cells (NPCs) and rod photoreceptors in vitro, then successfully implanted them into the site of cell damage. piPSCs not only incorporated into the host tissue at the site of implantation, but further extended beyond the grafted region long-term [54, 143]. The results suggest that piPSCs are capable of effectively integrating into host tissue, making them a candidate for clinical application.

### Equine

Two papers have been published describing an in vivo application of eiPSCs for the treatment of musculoskeletal injuries in equines [87, 144]. In the first paper, published in 2016, muscle injuries were induced in a GFP mouse model by injecting notexin, a myotoxic venom, along with an injection of eiPSCs. It was reported that these muscles saw an increase in myofiber production, and since eiPSCs were non-GFP reporting, it was shown that muscle fibers originating from the eiPSCs were produced. Undifferentiated cells remained in the muscle, indicating the dangerous potential for cancer formation [87].

To safeguard against potential cancer formation, another paper differentiated eiPSCs into MSCs prior to injection, reducing the risk of undesired proliferation. The eiPSC-MSCs were then injected into horses with various musculoskeletal disorders including fractures, tendonitis, osteochondrosis, and osteoarthritis. Improvements were observed including reduced lameness fever and fracture lines, although some horses also experienced hot flush and edema [144]. Although successful, this paper indicates a need for further development of less immune-reactive therapies.

Host immune responses are a major concern for clinical use of iPSCs, especially in species like horses where allogeneic cell use would be ideal. Further to the example above, another paper tested the immune potential of in vivo transplantation of allogeneic eiPSCs. Injected cells induced a minor, focal inflammatory response, but cellular signs of chronic inflammation persisted until the end of the study period 30 days after grafting. Undifferentiated cells have reduced expression of MHC surface proteins, but upon differentiation in vivo, these proteins increase, stimulating an increased immune reaction [145]. Although these cells were undifferentiated, the risk of immune response is significant and must still be addressed in differentiated eiPSCs, especially if this response increases with differentiation prior to implantation.

### Canine

Fewer developments have been made in ciPSC research, but a 2011 paper showed the potential for ciPSCs to be used for ischemic tissue damage treatment, both in hind limb ischemia and cardiac infarction mouse models [92]. ciPSCs were differentiated into endothelial cells (ciPSC-ECs), then injected into mice models. In hindlimb ischemia mice, ciPSC-ECs were shown to significantly improve revascularization in the compromised tissue. In cardiac infarction models, ciPSC-ECs were shown to engraft onto the heart muscle itself and improve cardiac contractility. In both models it was demonstrated that donor cells were lost over time, indicating a possible need for repeated treatments. Nevertheless, the lack of recurring original symptoms of ischemia suggests the capability of these cells to induce long-lasting, persistent effects in tissues following their disappearance [92].

### Barriers

#### Safety

There are several concerns to be resolved before in vivo use of iPSCs can be justified. Immune reactivity is one concern in the use of allogenic cells that has been discussed earlier in this view. Aside from the formation of undesired cell types, the most significant risk is in vivo tumorigenesis due to the proliferative potential of iPSCs. Current research has demonstrated that differentiation of iPSCs and purification of differentiated cellular products prior to implantation can reduce tumour formation [146]. Alternatively, tumour formation has been addressed in mice models with the application of “suicide genes”. Using a drug-inducible suicide system, apoptosis of iPSC-derived cells can be initiated with exposure to a particular drug [147]. This system would allow for the complete inactivation of iPSC derivatives in the event of aberrant growth or modification.

Immune reaction to transplanted iPSCs is another safety concern for clinical application. The use of autologous transplants would mitigate these effects, although is not realistic for commercialization of treatments due to prohibitively high costs. Research is being conducted into a cellular “cloaking” system that would allow cells to go undetected by the immune system of the host. Modification of allogeneic cells by altering MHC and HLA antigens has also shown potential to eliminate immune reaction in human iPSCs xenotransplantation studies [148]. However, this system is not without limitations; it is dangerous to create cell populations that cannot be controlled by the host immune system. Introducing a system that combines the drug-inducible suicide system and the cloaking system could potentially resolve this issue.

Many transduction systems used for iPSC production have inherent safety concerns due to their random integration into the host genome. Random integration can lead to disruptions in host genes and an increased risk of oncogenicity. A non-genome integrating Sendai virus system [149, 150] allows for the production of transgene-free iPSCs while maintaining a high reprogramming efficiency [149]. Similarly, a non-viral system that operates on the use of *piggyBac* transposons can create transgene-free iPSCs via excision from the genome following iPSC generation [151]. Originally described in human models, these systems have since been applied to domestic species, including dogs [88, 89], chickens [20], and cattle [152]. The wide range of reprogramming system options is beyond the scope of this article but have been reviewed elsewhere [153–155].

#### Technical barriers

Researchers have relied on precedent methods of human and mouse models to generate iPSCs in domestic animals [49, 114, 156]. An issue seen in many domestic models is the retention of pluripotent transgene expression; a situation that allows for the maintenance of pluripotency, or in many cases a pseudo-pluripotent state, that can interfere with differentiation. Most iPSCs derived from domestic species have been generated by viral integration of human or murine reprogramming transgenes that remain expressed [82, 83, 85, 86, 91]. The continuous expression of these transgenes suggests an incomplete epigenetic remodeling with OKSM factors alone and a greater need for understanding and optimizing the pluripotency induction process in domestic species. The use of non-viral vectors may prove effective in iPSC production, while overcoming the issue of transgene expression. Unfortunately, there is limited research on the application of iPSCs for disease research with the use of non-viral vectors. Yu and colleagues remain the only research group to successfully generate iPSCs using non-viral minicircles capable of generating chimeric chicks [20]. As few researchers have confirmed pluripotency by means of chimerism, confirmation of bona fide iPSCs has been limited. Often, cells believed to be iPSCs are iPSC-like cells as there are technical difficulties in yielding bona fide iPSCs that can maintain pluripotency independent of doxycycline [80]. Bona fide iPSCs remain difficult to obtain and further investigation into true iPSC production is required.

Despite significant species conservation of pluripotency genes, some divergence of the core pluripotency genes have been identified between mammals [157]. For example, the use of OSKM, Lin-28 and Nanog has been well established in porcine models, while other species are still under investigation, e.g. felids where OSKM plus NANOG may be required [23, 158]. It may be necessary to modify existing methods of achieving pluripotency, such as including additional reprogramming factors or developing different culture conditions to overcome species-specific reprogramming barriers.

#### Cost

Cost is another barrier to the application of iPSCs in domestic species due to laborious production. As previously mentioned, iPSCs from domestic animals have technical barriers limiting yield. As a result, more reagents, tissue, time, and labour are required for sufficient production [159]. Further costs have been associated with autologous iPSC treatment due to the production and maintenance of many cell lines and associated labour costs.

#### Future directions

Organoids, three-dimensional cell cultures that demonstrate characteristic development, anatomy, and physiology of a tissue, are currently an undeveloped tool in iPSCs derived from domestic species. Organoids have recently been developed from human iPSCs, which suggests the possibility of producing any organ of the body under the appropriate conditions. The use of iPSCs derived from domestic animals for organoid production could also be applied to veterinary medicine. Similar to human iPSCs, two-dimensional models have limitations in drug screen and assessing disease progression as they do not resemble in vivo conditions like organoids. Hence, virologists and drug developers can use them to better understand the mechanisms of disease or drug actions [160]. Several researchers have already exemplified the use of human embryonic stem cell-derived organoids in detecting harmful effects of toxins on the functionality and morphology of the organoids [161, 162], and the ability of organoids to be derived from tumorigenic tissue for drug testing [163]. Nevertheless, these studies have not yet been investigated in iPSC-derived organoids.

**Fig. 3:**
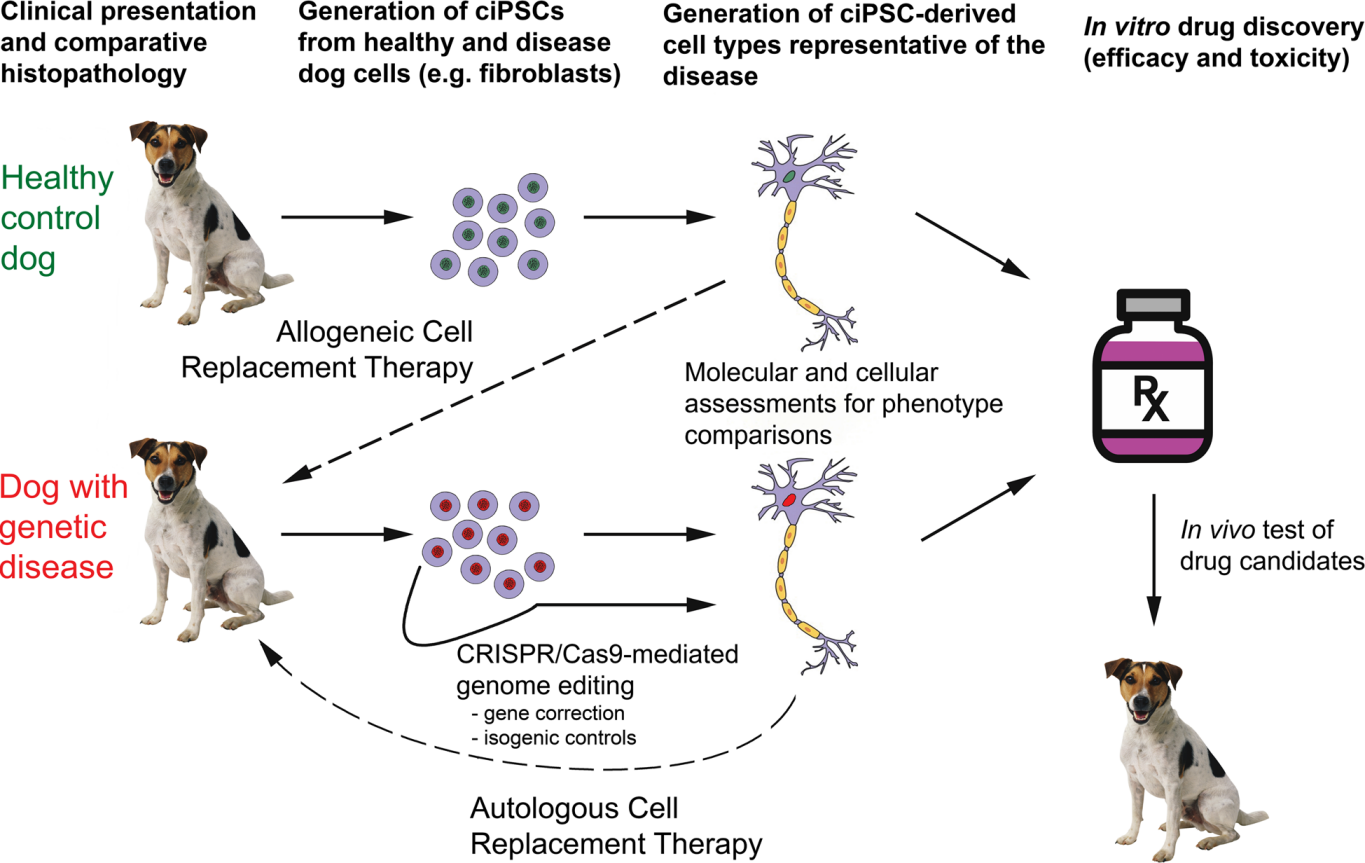
Potential Use of Domestic Animal iPSCs for Drug Discovery, Disease Modelling and Cell Replacement Therapy. Induced pluripotent stem cell (iPSCs) can be generated from healthy animals (e.g. dogs) for allogeneic cell transplantation of therapeutic cell types/tissue indicative of the disease. Alternatively, iPSCs can be generated from animals harbouring a genetic disorder and through CRISPR/Cas9-mediated genome editing technologies these genetic mutations can be corrected so that differentiated cell products from these iPSCs can be utilized in autologous cell replacement therapies. In addition, both the genetically mutated iPSCs and their genome-corrected iPSCs can be compared and contrasted for disease modelling purposes. This disease-in-a-dish could be potentially use as a high throughput screening system to discover novel drug candidates. Figure by Dean H. Betts (Adobe Photoshop)

CRISPR/Cas9 (clustered regularly interspaced short palindromic repeats-associated protein 9) mediated gene editing has been applied to iPSC research to correct or induce genetic mutations in iPSC lines, primarily in the study of monogenic diseases. Figure 3 demonstrates the potential research and clinical applications of CRISPR/Cas9-edited iPSCs in domestic species. Extensive research has been done in human models and has been reviewed previously [164, 165]. Although the use of genome editing technologies has been limited in domestic species, a single report of successful CRISPR editing of bovine iPSCs has been published [166]. Genome editing of iPSCs in combination with chimera generation provide the potential for transgenic animal development. In agricultural animals, the artificial introduction of valuable traits e.g. therapeutic proteins in milk, decreased waste product, and disease resistance, could be invaluable to the farming industry [20, 167]. Economically, this would require germline transmission, which has seen little success in domestic species as compared to rodents [20, 116, 168]. Further research is required to fully understand the feasibility, safety and ethical implications of germline transmission of genetically modified iPSCs from domestic species.

Proteomic, metabolic, and methylomic analysis of iPSCs have limited acknowledgement and investigation in research. However, there have been recent efforts to investigate the proteins and sites of methylation of human iPSCs and their derivatives. The investigation of -omics in iPSCs can assist in confirming how completely iPSCs have been reprogrammed to an undifferentiated stem cell state and resemble ESCs. Studying proteins and sites of methylation have clinical applications in autologous cell replacement therapy [169]. Aside from one study investigating the effects of epigenetic modifiers on silencing on exogenous transcription in piPSCs [65], epigenetics is an unexplored area of iPSC research in domestic species. In humans, research has shown isogenic iPSC populations and similar epigenetic markers of hiPSCs and hESCs [170]. Such results further emphasize the potential applicability of iPSCs in disease research and as a substitute for ESCs.

Although there is a steadily growing number of publications pertaining to porcine, equine, and canine models, the numbers are much fewer for cattle, goats, chickens, and cats. Hence, new research initiatives should further investigate these species for their potential application in the fields of disease modelling, treatment, and enhancement of production animals.

## Conclusion

Induced pluripotent stem cells are an innovative tool that hold great potential in contributing to veterinary medicine. Protocols for the production of iPSCs in some domestic species have been well-defined and have prompted research into their many potential applications. iPSC cultures have allowed for the production of tissues that can be studied for their physiological use and disease pathologies. Further, iPSCs themselves may be used in the future for the treatment of various diseases seen by veterinary practitioners. Although achievements have been made, a great deal of work is still required before these techniques can be clinically applied.

## Data Availability

All data analyzed during this study is included in this published article.

## Abbreviations

biPSCs: Bovine induced pluripotent stem cells
ciPSCs: Canine induced pluripotent stem cells
ciPSCs-ECs: Canine induced pluripotent stem cells differentiated into endothelial cells
CRISPR/Cas9: Clustered regularly interspaced short palindromic repeats-associated protein 9
EDCs: Endocrine disrupting compounds
eiPSCs: Equine induced pluripotent stem cells
eiPSC-KCs: Equine induced pluripotent stem cells differentiated into keratinocytes
ESCs: Embryonic stem cells
giPSCs: Galline induced pluripotent stem cells
iPSCs: Induced pluripotent stem cells
MSCs: Mesenchymal stromal cells
MVEV: Murray Valley encephalitis virus
NDV: Newcastle disease virus
NPCs: Neural progenitor cells
OSKM: Oct4, Sox2, Klf4, and c-Myc
piPSCs: Porcine induced pluripotent stem cells
VSMCs: Vascular smooth muscle cells
WNV: West Nile virus
